# Novel algorithmic approach predicts tumor mutation load and correlates with immunotherapy clinical outcomes using a defined gene mutation set

**DOI:** 10.1186/s12916-016-0705-4

**Published:** 2016-10-25

**Authors:** Jason Roszik, Lauren E. Haydu, Kenneth R. Hess, Junna Oba, Aron Y. Joon, Alan E. Siroy, Tatiana V. Karpinets, Francesco C. Stingo, Veera Baladandayuthapani, Michael T. Tetzlaff, Jennifer A. Wargo, Ken Chen, Marie-Andrée Forget, Cara L. Haymaker, Jie Qing Chen, Funda Meric-Bernstam, Agda K. Eterovic, Kenna R. Shaw, Gordon B. Mills, Jeffrey E. Gershenwald, Laszlo G. Radvanyi, Patrick Hwu, P. Andrew Futreal, Don L. Gibbons, Alexander J. Lazar, Chantale Bernatchez, Michael A. Davies, Scott E. Woodman

**Affiliations:** 1 Department of Melanoma Medical Oncology, The University of Texas MD Anderson Cancer Center, 1515 Holcombe Blvd., Unit 904, Houston, TX 77030 USA; 2 Department of Genomic Medicine, The University of Texas MD Anderson Cancer Center, Houston, TX 77030 USA; 3 Department of Surgical Oncology, The University of Texas MD Anderson Cancer Center, Houston, TX 77030 USA; 4 Department of Biostatistics, The University of Texas MD Anderson Cancer Center, Houston, TX 77030 USA; 5 Department of Pathology, The University of Texas MD Anderson Cancer Center, Houston, TX 77030 USA; 6 Department of Bioinformatics and Computational Biology, The University of Texas MD Anderson Cancer Center, Houston, TX 77030 USA; 7 Lion Biotechnologies, Woodland Hills, CA 91637 USA; 8 Department of Investigational Cancer Therapeutics, The University of Texas MD Anderson Cancer Center, Houston, TX 77030 USA; 9 Sheikh Khalifa Bin Zayed Al Nahyan Institute for Personalized Cancer Therapy, The University of Texas MD Anderson Cancer Center, Houston, TX 770393 USA; 10 Department of Systems Biology, The University of Texas MD Anderson Cancer Center, Houston, TX 77030 USA; 11 Department of Cancer Biology, The University of Texas MD Anderson Cancer Center, Houston, TX 77030 USA; 12 EMD Serono, Rockland, MA 02370 USA; 13 Department of Thoracic Medical Oncology, The University of Texas MD Anderson Cancer Center, Houston, TX 77030 USA

**Keywords:** Melanoma, Lung cancer, Total mutation load, CTLA-4, PD-1, Immunotherapy

## Abstract

**Background:**

While clinical outcomes following immunotherapy have shown an association with tumor mutation load using whole exome sequencing (WES), its clinical applicability is currently limited by cost and bioinformatics requirements.

**Methods:**

We developed a method to accurately derive the predicted total mutation load (PTML) within individual tumors from a small set of genes that can be used in clinical next generation sequencing (NGS) panels. PTML was derived from the actual total mutation load (ATML) of 575 distinct melanoma and lung cancer samples and validated using independent melanoma (*n* = 312) and lung cancer (*n* = 217) cohorts. The correlation of PTML status with clinical outcome, following distinct immunotherapies, was assessed using the Kaplan–Meier method.

**Results:**

PTML (derived from 170 genes) was highly correlated with ATML in cutaneous melanoma and lung adenocarcinoma validation cohorts (R^2^ = 0.73 and R^2^ = 0.82, respectively). PTML was strongly associated with clinical outcome to ipilimumab (anti-CTLA-4, three cohorts) and adoptive T-cell therapy (1 cohort) clinical outcome in melanoma. Clinical benefit from pembrolizumab (anti-PD-1) in lung cancer was also shown to significantly correlate with PTML status (log rank *P* value < 0.05 in all cohorts).

**Conclusions:**

The approach of using small NGS gene panels, already applied to guide employment of targeted therapies, may have utility in the personalized use of immunotherapy in cancer.

**Electronic supplementary material:**

The online version of this article (doi:10.1186/s12916-016-0705-4) contains supplementary material, which is available to authorized users.

## Background

There is a strong impetus to personalize the care of cancer patients to deliver selective therapies. One way this is being done clinically is by performing next-generation sequencing (NGS) on panels of cancer-related genes to identify targeted therapy approaches to which patients are most likely to respond. While a number of ongoing targeted therapy trials are utilizing this approach to optimize patient selection, in parallel, new immunotherapies have shown remarkable anti-cancer effects in melanoma and multiple other malignancies [1–6]. Notably, several recent whole exome sequencing (WES) studies have demonstrated a significant correlation between the total mutation load (i.e., the complete set of genes harboring non-synonymous, exonic mutations in a tumor) and clinical benefit with immune checkpoint inhibitors [7–9]. Widespread access to WES remains limited due to infrastructure requirements, cost and bioinformatic demands. The development of a method to accurately estimate total mutation load from widely available NGS gene panels could further personalize the care of cancer patients by improving patient selection for immune-based therapies. In order to address this unmet clinical need, we developed an algorithm to generate a predicted total mutation load (PTML) from the mutation status of a tumor from a small set of cancer-related genes. Several cohorts of patients were used to determine the accuracy of PTML, and to assess its association with clinical outcomes in melanoma and lung cancer patients treated with immunotherapies.

## Methods

### Patient cohorts and clinical evaluation

Tumor samples were derived from patients enrolled on protocols reviewed and approved by the Institutional Review Board of The University of Texas MD Anderson Cancer Center. Informed consent was obtained from all patients. The ipilimumab treatment cohort from MD Anderson consisted of melanoma patients (*n* = 76) for whom our institutional gene mutation panel test was performed [10]. The adoptive T-cell therapy using ex vivo expanded tumor infiltrating lymphocytes (TIL) (ACT-TIL) cohort consisted of melanoma patients from MD Anderson (*n* = 36) for whom WES and/or institutional gene mutation panel testing was performed. All other sample data has been previously reported [9, 11–16]. Tumors were evaluated using standard cross-sectional imaging analysis according to study protocol or standard of care. The clinical outcomes of progression-free survival (PFS) and overall survival (OS) were determined from the date of initial treatment to the date of tumor progression and/or death (or loss to follow-up), respectively.

### Derivation of the ATML and PTML

The Cancer Genome Atlas (TCGA) mutation data from melanoma (SKCM) and lung adenocarcinoma (LUAD) samples were downloaded from the public TCGA data repository website of the Broad Institute (http://gdac.broadinstitute.org). The actual total mutation load (ATML) for each sample was considered as the sum of somatic, non-synonymous, exonic mutations of each gene, as derived from WES. A set of 170 cancer-related genes assayed at our institution (Additional file 1: Table S1) [10] were used to generate the cancer-specific PTMLs from the ATMLs of SKCM and LUAD TCGA samples [11, 17]. First, an unadjusted mutation value for each of the 170 genes in the panel was determined by identifying a TCGA sample with the lowest ATML in which the panel gene was mutated (Additional file 1: Figure S1A and B). Second, the sum of the unadjusted mutation values within a sample was compared to the ATML for that sample by regression analysis (Additional file 1: Figure S1C and D). Third, an adjusted mutation value was derived for each of the 170 genes using the slope of the line of the regression analysis (Additional file 1: Table S2 and S3). Finally, the PTML for each sample was generated by taking the sum of the adjusted mutation values.

### Derivation of the melanoma and lung cancer PTML

Derivation of the melanoma PTML was performed by initially determining an unadjusted gene mutation value for each of the 170 genes using the publicly available SKCM TCGA dataset (Additional file 1: Figure S1A). Any somatic mutation, regardless of “recurrent” or “non-recurrent” status, was included and silent mutations were excluded. Only one of the 170 genes in the panel was without a mutation in the 345 melanoma samples, and thus the remaining 169 genes were used for the algorithm. For each melanoma sample, the unadjusted individual gene mutation value for each of the 169 genes was determined by identifying the lowest ATML within each TCGA sample to harbor a mutation in one of the 169 genes in the panel. The sum of the unadjusted individual gene mutation values for each of the 169 genes defined the unadjusted total mutation load for a tumor sample. Regression analysis comparing the unadjusted total mutation load versus the ATML for each sample was highly correlated (*n* = 345, R^2^ = 0.87, Additional file 1: Figure S1C), from which the adjusted individual gene mutation value was then determined for each gene employing the slope of the regression line. Summation of each adjusted individual gene mutation value for each of the 169 genes within a sample provided the PTML for that sample. In cases in which a gene harbored more than one mutation, the adjusted individual gene mutation value was multiplied by the number of mutations within the gene. The same approach was taken to derive the lung cancer PTML using the LUAD TCGA dataset. Ten of the 170 genes in the panel were without a mutation in the 230 lung adenocarcinoma samples, and thus the remaining 160 genes were used for the algorithm. Regression analysis comparing the unadjusted total mutation load versus the ATML for each sample showed a strong correlation (*n* = 230, R^2^ = 0.76, Additional file 1: Figure S1D) from which the adjusted individual gene mutation values were determined for each gene. The following equation represents how to calculate the PTML:

$$ \boldsymbol{PTML} = {\displaystyle \sum_{k=1}^n}RND\left(\frac{MIN\left( total\  nonsynonymous\  exonic\  mutations\right)\ for\  gene\ k}{slope\  of\  linear\  regression}\right) $$

where, *n* = number of genes in the panel, RND denotes rounding, MIN denotes the minimum of total non-synonymous, exonic mutations among the samples with gene *k* mutated.

The adjusted individual gene mutation value for each gene differed substantially between the melanoma and lung adenocarcinoma. For example, a hotspot somatic mutation in *BRAF* (common in melanoma) equates to an adjusted gene mutation value of 7 if present within a melanoma sample, but garners a value of 22 if mutated in a lung adenocarcinoma sample (Additional file 1: Figure S1A and B). Inversely, a somatic mutation in *EGFR* (relatively common in lung cancer) equates to an adjusted gene mutation value of 7 for a lung adenocarcinoma sample, but has a value of 29 if present within a melanoma sample. This observation is consistent with the relative recurrent nature of these mutations within these cancer types.

### Statistical analyses

Kaplan–Meier analyses and the log-rank tests were performed using the ‘survival’ R package. Adjusted hazard ratios were determined using a Cox proportional hazards regression model. Median survival represents the time at which fractional survival equals 50 % (median_50_). If survival exceeds 50 % at the longest time point, then median survival is deemed “undefined.” The association of PTML status with serum lactate dehydrogenase (LDH) level was evaluated using the χ^2^ test, and with infused TIL number by unequal variance two-sample *t*-test.

## Results

### Derivation of PTML in melanoma and lung cancer samples

We sought to determine if mutations identified within a small, defined set of genes could be utilized to accurately predict the total mutation burden determined by WES. PTML algorithm development included 170 cancer-related genes that are part of our institutional assay panel (Additional file 1: Table S1) [10]. The mutation status of these genes and the ATML were identified for publicly-available WES mutation (somatic, non-synonymous, exonic) data from SKCM (*n* = 345) and LUAD (*n* = 230) samples derived from TCGA [10, 11, 17], and was used to develop and test our PTML algorithm (see Methods). The PTML value given to each gene, if mutated, differed substantially between melanoma and lung cancer, indicating differential association of the 170 cancer-related genes with the ATML in each cancer type (Additional file 1: Figure S1A and B; Table S2 and S3).

### Validation of PTML using multiple independent melanoma and lung cancer datasets

**Fig. 1:**
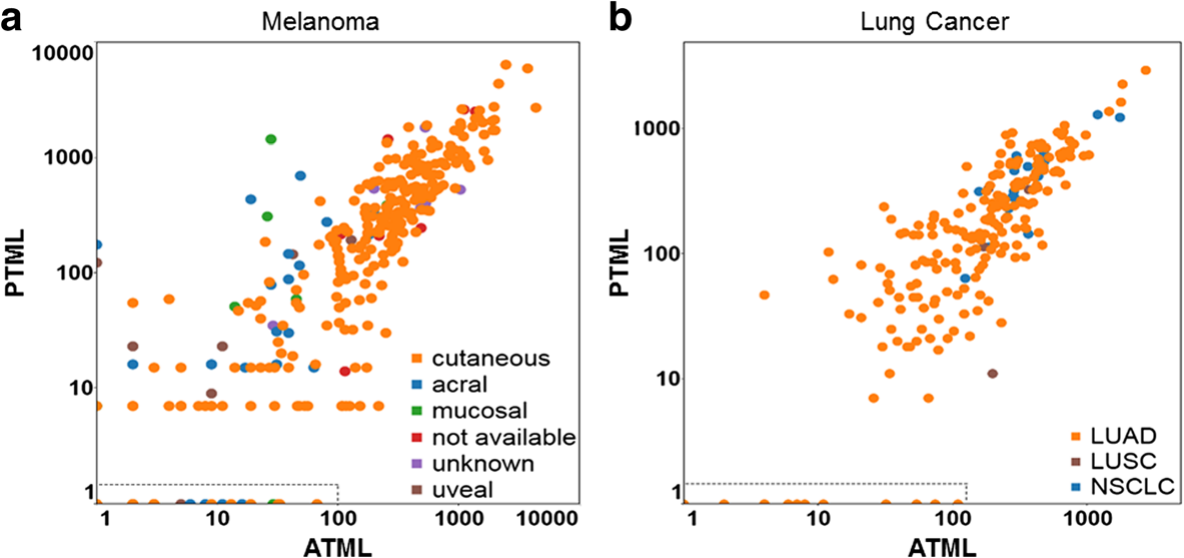
Melanoma and lung cancer have distinct predicted total mutation loads (PTMLs) that predict actual total mutation load (ATML). For validation, PTML was generated for independent melanoma cohorts (3 pooled cohorts, n = 312, R^2^ = 0.71; cutaneous only: *n* = 258, R^2^ = 0.73) [12–14] (**a**) and lung cancer (2 pooled cohorts, *n* = 217, R^2^ = 0.81; lung adenocarcinoma (LUAD) only: *n* = 199, R^2^ = 0.82) samples (**b**) [15, 16]. The melanoma- and lung-specific PTML strongly correlated with the ATML of samples from each cancer type, respectively. The lung cancer PTML performs well for both LUAD, lung squamous cell carcinoma, and not otherwise specified non-small cell lung carcinoma samples. A PTML score of zero correlates strongly with an ATML ≤ 100 in both cancers

In order to validate the melanoma PTML algorithm, regression analysis assessing the PTML versus ATML for melanoma samples from three (*n* = 121, *n* = 127, *n* = 64) independent, publicly available WES melanoma datasets was performed (Fig. 1a) [12–14]. The PTML was strongly correlated with the ATML from these melanoma validation cohorts (R^2^ = 0.71). Similarly, analysis of two independent cohorts of lung cancer (all NSCLC, 91 % adenocarcinoma) samples (*n* = 217) [15, 16] that had undergone WES also demonstrated a strong correlation between the lung cancer PTML and the ATML of these validation cohorts (R^2^ = 0.81, Fig. 1b). These data show that application of the cancer-specific PTMLs, derived from a small set of genes, to five unrelated validation cohorts accurately reflects the total mutation load within a tumor.

The correlation of adjusted gene mutation values for melanoma versus lung cancer was extremely poor (R^2^ = 0.0063, Additional file 1: Figure S2A). Further, application of a cancer-specific PTML to a distinctly different cancer dramatically weakened the correlation between the PTML and the ATML (R^2^ = 0.47, melanoma vs. lung cancer, Additional file 1: Figure S2B). In addition, a PTML value of zero significantly correlated with an ATML of less than 100 for both cancers (see dashed rectangle on *x*-axes in Fig. 1a, b), suggesting a predictive cut-off value when no PTML genes are mutated in a respective sample. Finally, the melanoma- and lung cancer-specific PTMLs derived from our 170 gene panel showed similar predictive value as the PTML derived from the most recurrently mutated genes that define each tumor type by WES, respectively (Additional file 1: Figure S3A and B).

### PTML in both melanoma and lung cancer correlates with nucleotide alterations associated with exposure to respective mutagens

**Fig. 2:**
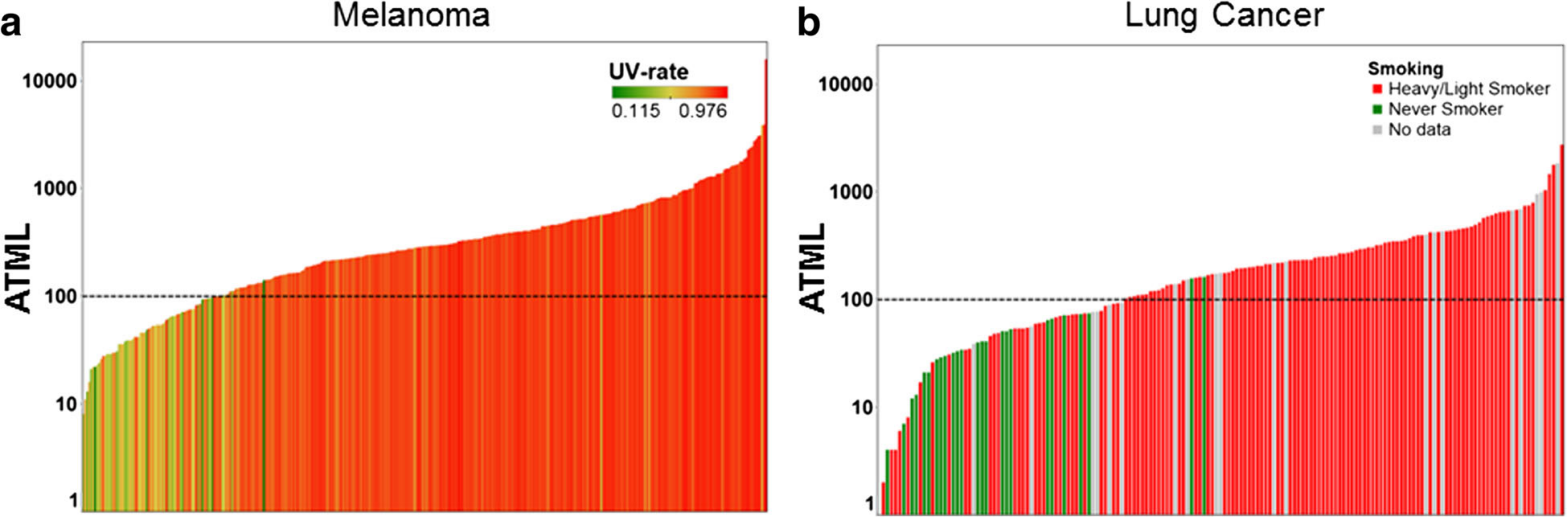
Melanoma and lung cancer-specific predicted total mutation loads correlate with specific mutagen exposures and nucleotide alterations. **a** Melanoma samples are ranked (x-axis) according to actual total mutation load (ATML) (y-axis), and the UV rate associated with each sample is displayed by colors (green = less exposure, red = more exposure). **b** Lung cancer samples are ranked (x-axis) according to ATML (y-axis), and the smoking status associated with each sample is displayed in color (green = never, red = smoker, gray = no data). Note the relative enrichment, but not complete constituency, of low UV-rate and never-smokers in less mutated samples, respectively. Samples were derived from the skin cutaneous melanoma (SKCM; *n* = 345) and lung adenocarcinoma (LUAD; *n* = 230) TCGA cohorts [11, 17]. LUSC, lung squamous cell carcinoma samples; NSCLC, not otherwise specified non-small cell lung carcinoma samples

The development of cutaneous melanoma and lung cancer is highly associated with exposure to distinct mutagens: ultraviolet (UV) radiation and tobacco smoking, respectively. Ranking melanoma samples from lowest to highest mutation load revealed a clear association between samples with lower mutation burdens and low UV rate (Fig. 2a). We observed a similar association for lung cancer and never-smoker status (Fig. 2b). Consistent with this observation, the melanoma- and lung cancer-specific PTMLs strongly correlated (R^2^ > 0.90) with the frequency of UV-mediated and tobacco-induced mutations (Additional file 1: Figure S4) [13, 15]. Notably, a distinct subset of samples, enriched for, but not exclusively composed of, low UV rate and never-smoker patient tumors, aggregated below the 100 total mutation threshold.

### Clinical utility of PTML in melanoma and lung cancer immunotherapy

It has been shown that the ATML of a tumor highly correlates with the frequency of potential neoantigens within that tumor [9, 18]. It is thought that the high ATML exhibited by melanoma and lung tumors increases the likelihood of generating neoantigens recognized by the immune system, and may play an important role in the efficacy of immunotherapy in these cancers [7, 8, 19]. However, as shown in the analysis in Fig. 2, a distinct subset of melanoma and lung cancer tumors harbor a low ATML (≤ 100), which has been shown to result in a much lower frequency of potential neoantigens [9, 18]. This is consistent with recent publications showing patients with melanoma tumors having ATMLs less than 100 to have significantly less clinical benefit from anti-CTLA-4 therapy [9, 14].

**Fig. 3:**
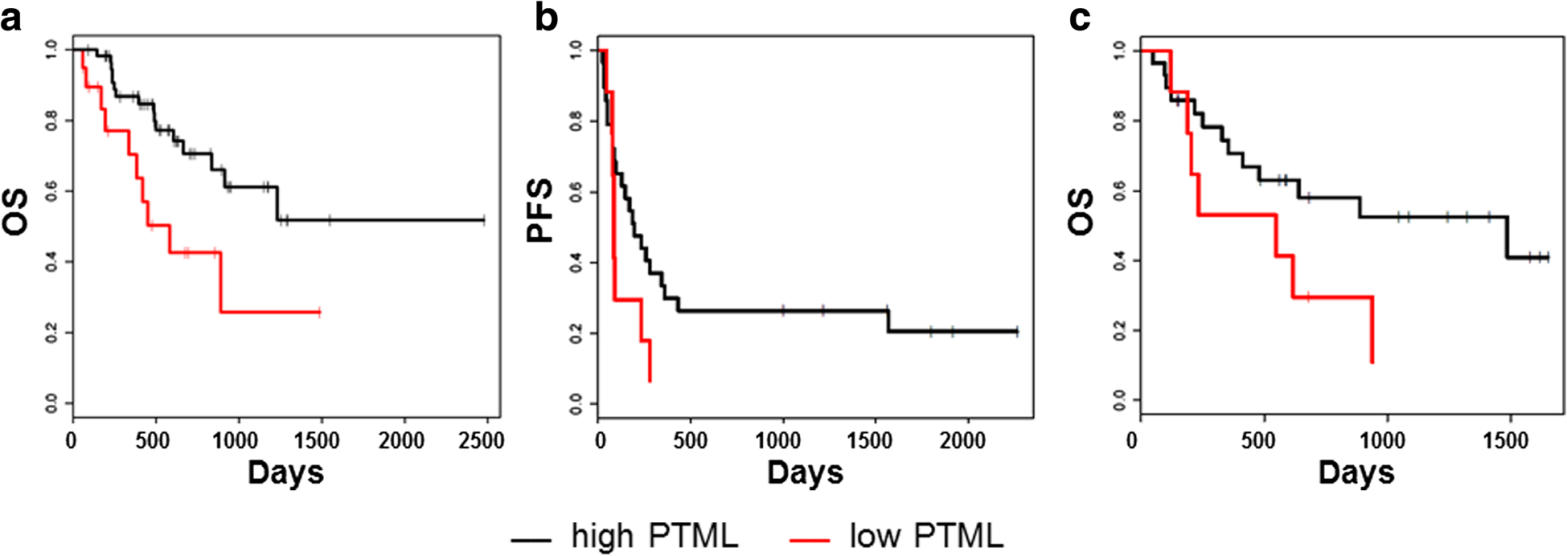
Predicted total mutation load (PTML) correlates with immunotherapy outcomes in melanoma. **a** Ipilimumab-treated melanoma patients with a low PTML (≤ 100, *n*= 19) have a poorer overall survival (OS) compared to high PTML patients (> 100, *n* = 57). Melanoma patients treated with adoptive T-cell therapy using ex vivo expanded tumor infiltrating lymphocytes with a low PTML (*n* = 8) have shorter progression-free survival (**b**) and shorter OS (**c**) compared to high PTML (*n* = 28) patients (low PTML = red line, high PTML = black line)

Since melanoma- and lung cancer-specific PTMLs significantly correlated with ATMLs and the characteristic nucleotide alterations within each cancer, we determined if the PTML also correlated with immunotherapy clinical outcomes in each cancer type. The PTML was first determined for a cohort of metastatic melanoma patients (*n* = 76) treated with the FDA-approved immunotherapy ipilimumab (anti-CTLA-4) (Additional file 1: Table S4). OS from the start of ipilimumab therapy was significantly shorter for patients with a low PTML (PTML ≤ 100, *n* = 19, median_50_ = 582 days) compared to patients with a higher PTML (PTML > 100, n = 57, median_50_ undefined, *P* = 0.006) (Fig. 3a). The unadjusted hazard ratio (HR) for low versus high PTML was 0.35 (95 % CI, 0.16–0.77; *P* = 0.009). After adjustment for LDH level, a known prognostic factor in metastatic melanoma that also correlates with inferior outcomes with immunotherapy in this disease [20], as well as for age, gender and clinical M stage, the HR for high PTML was 0.272 (95 % CI, 0.11–0.65; *P* = 0.003). The adjusted HR for elevated LDH was 4.45 (95 % CI, 1.93–10.8; *P* = 0.001). Furthermore, application of the PTML to two independent, previously reported cohorts of advanced melanoma patients treated with anti-CTLA-4 and for which WES data was available (*n* = 110 and *n* = 64) [9, 14] also demonstrated a strong association between the PTML and clinical benefit (Additional file 1: Figure S5A and B).

ACT-TIL is also an immunotherapy used in the treatment of melanoma [21]. We applied the PTML algorithm to an independent cohort of metastatic melanoma patients treated with ACT-TIL (*n* = 36) at our institution to assess the correlation between PTML and ACT-TIL clinical outcome (Additional file 1: Table S5). Patients with low PTML (*n* = 8) had significantly shorter PFS and OS than patients with high PTML (*n* = 28) (PFS: median_50_ 85 days vs. 190 days (*P* < 0.05); and OS: median_50_, 391 days vs. 1486 days (*P* < 0.05)) (Fig. 3b, c). Patients in these two groups did not significantly differ in the mean number of TILs administered (4.16 × 10^10^; SD = 1.98 × 10^10^ and 6.37 × 10^10^; SD = 3.58 × 10^10^, respectively, *P* = 0.11). The ACT-TIL cohort serum LDH level statistical analysis was inconclusive given the large confidence interval range, likely due to the limitations of the cohort size. No significant difference in median age (47.5 vs. 50.5 years old) or male to female ratio was observed between the low and high PTML patients.

**Fig. 4:**
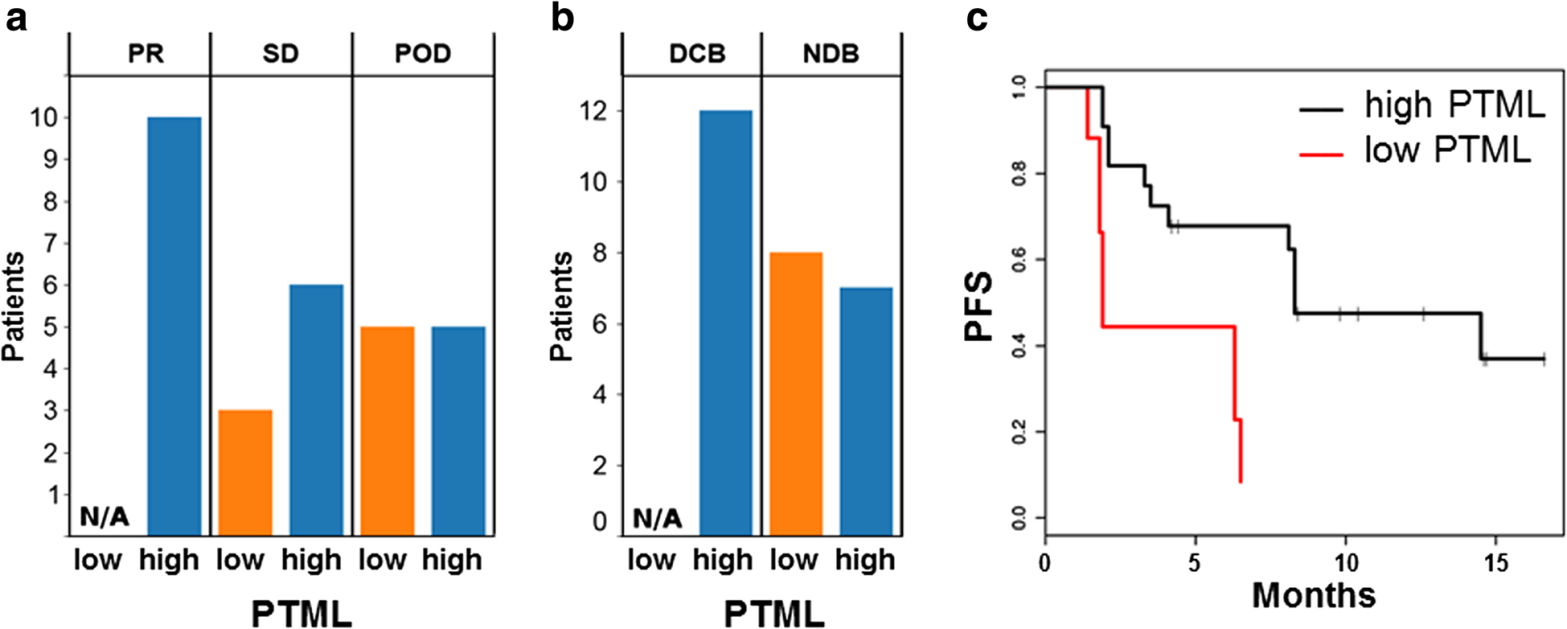
Predicted total mutation load (PTML) correlates with immunotherapy outcomes in lung adenocarcinoma. Lung adenocarcinoma patients with a low PTML (≤ 100) treated with pembrolizumab fail to obtain a partial tumor response (**a**), fail to obtain a durable clinical response (**b**), and have a markedly lower progression-free survival (PFS) compared to patients with a high PTML (**c**) (PFS curve: low PTML = red line, high PTML = black line)

Finally, PTML was determined for a previously reported cohort of metastatic lung adenocarcinoma patients treated with pembrolizumab (anti-PD-1) (Fig. 4a–c) [16]. As the observed distributions of PTMLs in the lung adenocarcinoma TCGA supported a threshold value of ≤ 100 non-synonymous mutations, clinical outcomes were analyzed by this threshold, which was also the same cut-off for the melanoma cohorts. None of the lung adenocarcinoma patients with low PTML (≤ 100; *n* = 8) achieved a clinical response (Fig. 4a) or durable clinical benefit (Fig. 4b), and they had a shorter PFS than patients with high PTML (*n* = 21) (4.1 vs. 8.3 months, *P* = 0.0003, Fig. 4c). Thus, the lung cancer-specific PTML recapitulated the clinical correlation results derived from the WES sample data, and the PTML ≤ 100 threshold separated a significant subset of patients with no clinical response/benefit in this cohort.

## Discussion

In this analysis, we employed WES data from 1104 distinct tumor samples to derive and validate an algorithm that accurately predicts the total mutation load for melanomas or lung cancers using results from a set of 170 genes broadly used in NGS cancer gene panels. We demonstrate that the individual genes were “weighted” differently depending on tumor type, creating melanoma- and lung cancer-specific PTML algorithms. These cancer-specific PTMLs strongly correlated with ATMLs from WES in both cancer types, and with clinical outcomes using three distinct immunotherapies (i.e., anti-CTLA-4, anti-PD-1, and ACT-TIL). These results suggest that small NGS mutation panels used to select targeted therapy approaches may also have utility for the personalized use of immunotherapies.

The relative cost comparison of targeted next generation sequencing of 169 genes versus WES of 20,000 genes depends on the assessment of multiple variables: the depth of sequencing, extra reagent expense, difference in flow cell occupancy, contrast in informatics and data storage needs, and time costs. We estimate this difference to be at least 5- to 10-fold greater to perform targeted next generation sequencing on approximately 200 gene exomes versus WES with current technology and resources.

The clinical benefit observed in melanoma and lung cancer to single-agent immunotherapy is thought to be related to these two tumor types having the highest frequency of gene mutations among common solid cancers, and thus increasing the likelihood of generating neoantigens recognized by the immune system [7, 8, 19]. Multiple WES studies have now shown a significant correlation between the total tumor mutation load and the predicted neoantigen load [9, 14, 18, 22]. In addition, recent data from the melanoma and lung adenocarcinoma TCGAs showed a strong association between total tumor mutation load and immune cytolytic activity within samples, with an abrupt increase in the immune cytolytic activity within both tumor types beginning at approximately 100 total mutations within a tumor [9, 18]. Consistent with this data, a recent study by Van Allen et al. [9] showed, in an independent melanoma cohort, that both the mutation/neoantigen load and the expression of immune cytolytic enzymes are associated with clinical benefit. Our own analysis of this data indicates that the combination of mutation load and cytolytic score results in an even more significant correlation with clinical benefit (unpublished data).

In this study, we observed that patients with a low PTML (≤ 100) had worse clinical outcomes compared to patients with a high PTML (> 100) in three independent advanced melanoma cohorts treated with ipilimumab. We also report here, for the first time, that low PTML is associated with significantly shorter PFS and OS in a cohort of metastatic melanoma patients treated with ACT-TIL. The correlation between ACT-TIL clinical outcome and the total mutation load is reinforced by prior case reports suggesting that benefit from ACT-TIL may be due to the existence and persistence of clones within the TIL that recognize neoantigens on the matched cancer cells [23, 24]. Finally, utilizing the PTML ≤ 100 threshold, patients with lung adenocarcinoma and a low PTML failed to achieve partial tumor response, failed to achieve durable clinical benefit, and had a lower PFS. Thus, low PTML represented a clinically worse outcome category in pembrolizumab-treated lung adenocarcinoma.

Despite the association between tumor mutation load and clinical outcome in these and other studies, as with other effective markers in cancer, the relationship between these variables is not deterministic. Patients with melanoma or lung cancer that harbor a “low” tumor mutation load can “respond” to immunotherapy and those with a “high” mutation load may not respond. However, our analysis across five clinical data sets in two cancer types indicates that, below a threshold level, the probability of clinical benefit is significantly lower. Thus, the total mutation load or the PTML may serve as an important variable when assessing the potential benefits of immune-based therapeutics in individual patients. In addition, the PTML demonstrated efficacy across cohorts in which distinct tissue procurement, exome capture/sequencing techniques, mutation calling algorithms, and different definitions of clinical benefit were used. In this study, we were agnostic about the specific location and functionality of a gene mutation and its association with the ATML. Further studies to enhance the use of the PTML could focus on standardizing analytic practices, potentially assigning further weight to functionally relevant genes/mutation sites and combining the PTML with other variables that may have predictive value (i.e., immune infiltrate or cytolytic score) as has been suggested [9].

There are multiple examples of oncogenic mutations that predict benefit from FDA-approved targeted therapies (CML/imatinib, melanoma/BRAFi, lung/EGFR and ALK inhibitors, breast/HER2 inhibitors), and other mutations that predict resistance (*RAS* mutations and EGFR inhibitors). Taken together, these data support the clinical use of molecular testing to guide personalized cancer treatment [25]. In addition, multiple trials are currently ongoing in which patients are assigned to investigational targeted therapy strategies based on the results of clinical NGS panels. Here, we demonstrate that the mutation status of a small set of genes, that could also be used to select rationale targeted therapies, provides an accurate estimate of the total mutation load (PTML) which significantly correlates with clinical benefit from immunotherapy in melanoma and lung cancer patients. These results provide a new and easily actionable approach to personalize the care of cancer patients and to further optimize the use of immune therapies.

## Conclusions

We have developed an innovative algorithmic approach using large cohorts of melanoma and lung cancer samples with DNA sequencing data that can predict the total mutation load from a set of less than 170 genes. The predicted total mutation load from this limited gene set correlates significantly with immunotherapy outcomes in melanoma and lung cancer and may have significant utility in the personalized use of immunotherapy in advanced cancer patients.

## Additional file

Additional file 1: Supplementary methods, tables, and figures. (DOCX 646 kb)

## Abbreviations

ATML: actual total mutation load
LUAD: lung adenocarcinoma
NGS: next generation sequencing
OS: overall survival
PFS: progression-free survival
PTML: predicted total mutation load
SKCM: skin cutaneous melanoma
WES: whole exome sequencing
