# Supplementary material for: Novel algorithmic approach predicts tumor mutation load and correlates with immunotherapy clinical outcomes using a defined gene mutation set
Source: BMC Med. 2016 Oct 25;14:168. doi: 10.1186/s12916-016-0705-4 (PMC5078889; doi:10.1186/s12916-016-0705-4)
Supplement: Additional file 1: — Supplementary methods, tables, and figures. (DOCX 646 kb) [file 12916_2016_705_MOESM1_ESM.docx]

**Additional file 1**

This appendix has been provided by the authors to give readers additional information about their work.

**Novel algorithmic approach predicts tumor mutation load and correlates with immunotherapy clinical outcomes using a defined gene mutation set**

Jason Roszik, PhD, Lauren E. Haydu, MIPH, Kenneth R. Hess, PhD, Junna Oba, MD, PhD, Aron Y. Joon, MS, Alan E. Siroy, MD, Tatiana V. Karpinets, PhD, DSc, Francesco C. Stingo, PhD, Veera Baladandayuthapani, PhD, Michael T. Tetzlaff, MD, PhD, Jennifer A. Wargo, MD, MMSc, Ken Chen, PhD, Marie-Andrée Forget, PhD, Cara L. Haymaker, PhD, Jie Qing Chen, PhD, Funda Meric-Bernstam, MD, Agda K. Eterovic, PhD, Kenna R. Shaw, PhD, Gordon B. Mills, MD, PhD, Jeffrey E. Gershenwald, MD, Laszlo G. Radvanyi, PhD, Patrick Hwu, MD, P. Andrew Futreal, PhD, Don L. Gibbons, MD, PhD, Alexander J. Lazar, MD, PhD, Chantale Bernatchez, PhD, Michael A. Davies, MD, PhD, Scott E. Woodman, MD, PhD

**Corresponding authors:**

Jason Roszik ([jroszik@mdanderson.org](mailto:jroszik@mdanderson.org))

Scott E. Woodman ([swoodman@mdanderson.org](mailto:swoodman@mdanderson.org))

**Table of contents:**

1. **Supplementary Methods**
2. **Supplementary Tables**

Table S1. Set of 170 cancer-related genes assayed by our cancer center (and others) and commercial organizations.

Table S2. *Adjusted* gene mutation value for each gene for melanoma

Table S3. *Adjusted* gene mutation value for each gene for lung cancer

Table S4. Melanoma cohort treated with ipilimumab (n=76)

Table S5. Melanoma cohort treated with ACT-TIL (n=36)

1. **Supplementary Figures**

Figure S1. Derivation of the PTML

Figure S2. Cancer Specificity of PTML

Figure S3. Total mutation burden predicted using recurrently mutated genes

Figure S4. PTML correlates with characteristic nucleotide types

Figure S5. Low PTML correlates with poorer clinical results in anti-CTLA-4 treated melanoma tumors

1. **Supplementary References**
2. **Supplementary Methods**

**Sample Cohorts**: Samples for the ipilimumab treatment cohort were procured under MD Anderson protocols (PA11-0852 and 2004-0069). Not included in the analysis of the ipilimumab treatment cohort were patients who received adjuvant immunotherapy; biochemotherapy, high dose IL2 or ACT-TIL immune therapies. LDH levels were those taken most proximal to treatment, elevated LDH was defined as above the upper limit of normal. Samples for the ACT-TIL treatment cohort were procured under MD Anderson clinical protocol NCT00338377. The following accession number are provided: Hodis et al [1] (dbGaP accession number: phs000452.v1.p1); Krauthammer et al [2] (from Supplementary Text and Figures); Snyder et al [3] (from Supplementary Appendix); Imielinski et al [4] (Supplementary file Table S4, dbGAP accession: phs000488.v1.p1); Rizvi et al [5] (dbGAP accession: phs000980.v1.p1).

1. **Supplementary Tables**

**Table S1. Set of 170 cancer-related genes assayed by our cancer center (and others) and commercial organizations**

| *ABL1* | *CD79A* | *FAM123B* | *KEAP1* | *NRAS* | *SMARCB1* |
| --- | --- | --- | --- | --- | --- |
| *AKT1* | *CD79B* | *FANCA* | *KIT* | *NTRK1* | *SMO* |
| *AKT2* | *CDC73* | *FANCD2* | *KRAS* | *NTRK3* | *SOCS1* |
| *AKT3* | *CDH1* | *FBXW7* | *LRP1B* | *PALB2* | *SPEN* |
| *ALK* | *CDK12* | *FGFR1* | *MAP2K1* | *PAX5* | *SPOP* |
| *APC* | *CDK4* | *FGFR2* | *MAP2K2* | *PBRM1* | *SRC* |
| *AR* | *CDK6* | *FGFR3* | *MAP2K4* | *PDGFRA* | *STAG2* |
| *ARAF* | *CDKN1B* | *FGFR4* | *MAP3K1* | *PDGFRB* | *STK11* |
| *ARID1A* | *CDKN2A* | *FLT1* | *MCL1* | *PIK3CA* | *SUFU* |
| *ARID2* | *CDKN2C* | *FLT3* | *MDM2* | *PIK3CG* | *TET2* |
| *ASXL1* | *CEBPA* | *FLT4* | *MED12* | *PIK3R1* | *TGFBR2* |
| *ATM* | *CHEK1* | *FOXL2* | *MEN1* | *PPP2R1A* | *TNFAIP3* |
| *ATR* | *CHEK2* | *GATA1* | *MET* | *PRDM1* | *TOP1* |
| *ATRX* | *CIC* | *GATA2* | *MITF* | *PTCH1* | *TP53* |
| *AURKA* | *CREBBP* | *GATA3* | *MLH1* | *PTEN* | *TSC1* |
| *AURKB* | *CSF1R* | *GNA11* | *MLL* | *PTPN11* | *TSC2* |
| *AXL* | *CTCF* | *GNAQ* | *MLL2* | *RAD51* | *TSHR* |
| *BAP1* | *CTNNB1* | *GNAS* | *MPL* | *RAF1* | *VHL* |
| *BCL2* | *DAXX* | *GSK3B* | *MSH2* | *RARA* | *WT1* |
| *BCOR* | *DDR2* | *HRAS* | *MSH6* | *RB1* | *XPO1* |
| *BRAF* | *DNMT3A* | *IDH1* | *MTOR* | *RET* |  |
| *BRCA1* | *EGFR* | *IDH2* | *MUTYH* | *RICTOR* |  |
| *BRCA2* | *EP300* | *IGF1R* | *MYD88* | *RNF43* |  |
| *BTK* | *EPHA3* | *IL7R* | *NF1* | *RPTOR* |  |
| *CARD11* | *EPHA5* | *JAK1* | *NF2* | *RUNX1* |  |
| *CBL* | *ERBB2* | *JAK2* | *NFE2L2* | *SETD2* |  |
| *CCND1* | *ERBB3* | *JAK3* | *NKX2-1* | *SF3B1* |  |
| *CCND2* | *ERBB4* | *KDM5C* | *NOTCH1* | *SMAD2* |  |
| *CCND3* | *ESR1* | *KDM6A* | *NOTCH2* | *SMAD4* |  |
| *CCNE1* | *EZH2* | *KDR* | *NPM1* | *SMARCA4* |  |

**Table S2. *Adjusted* gene mutation value for each gene for melanoma**

| ***ABL1*** | 68 | ***CDK4*** | 29 | ***FLT3*** | 19 | ***MITF*** | 167 | ***RAF1*** | 88 |
| --- | --- | --- | --- | --- | --- | --- | --- | --- | --- |
| ***AKT1*** | 122 | ***CDK6*** | 36 | ***FLT4*** | 102 | ***MLH1*** | 244 | ***RARA*** | 117 |
| ***AKT2*** | 88 | ***CDKN1B*** | 845 | ***FOXL2*** | 1632 | ***MLL*** | 29 | ***RB1*** | 44 |
| ***AKT3*** | 197 | ***CDKN2A*** | 28 | ***GATA1*** | 377 | ***MLL2*** | 68 | ***RET*** | 82 |
| ***ALK*** | 55 | ***CDKN2C*** | 418 | ***GATA2*** | 54 | ***MPL*** | 114 | ***RICTOR*** | 122 |
| ***APC*** | 16 | ***CEBPA*** | 1473 | ***GATA3*** | 217 | ***MSH2*** | 155 | ***RNF43*** | 55 |
| ***AR*** | 334 | ***CHEK1*** | 226 | ***GNA11*** | 23 | ***MSH6*** | 148 | ***RPTOR*** | 114 |
| ***ARAF*** | 148 | ***CHEK2*** | 21 | ***GNAQ*** | 9 | ***MTOR*** | 15 | ***RUNX1*** | 515 |
| ***ARID1A*** | 59 | ***CIC*** | 19 | ***GNAS*** | 41 | ***MUTYH*** | 71 | ***SETD2*** | 32 |
| ***ARID2*** | 40 | ***CREBBP*** | 63 | ***GSK3B*** | 690 | ***MYD88*** | 385 | ***SF3B1*** | 9 |
| ***ASXL1*** | 116 | ***CSF1R*** | 161 | ***HRAS*** | 176 | ***NF1*** | 25 | ***SMAD2*** | 247 |
| ***ATM*** | 34 | ***CTCF*** | 377 | ***IDH1*** | 23 | ***NF2*** | 99 | ***SMAD4*** | 59 |
| ***ATR*** | 11 | ***CTNNB1*** | 40 | ***IDH2*** | 900 | ***NFE2L2*** | 294 | ***SMARCA4*** | 131 |
| ***ATRX*** | 55 | ***DAXX*** | 153 | ***IGF1R*** | 20 | ***NKX2-1*** | 342 | ***SMARCB1*** | 87 |
| ***AURKA*** | 125 | ***DDR2*** | 76 | ***IL7R*** | 21 | ***NOTCH1*** | 34 | ***SMO*** | 211 |
| ***AURKB*** | 156 | ***DNMT3A*** | 130 | ***JAK1*** | 125 | ***NOTCH2*** | 21 | ***SOCS1*** | 687 |
| ***AXL*** | 76 | ***EGFR*** | 29 | ***JAK2*** | 106 | ***NPM1*** | 76 | ***SPEN*** | 64 |
| ***BAP1*** | 122 | ***EP300*** | 114 | ***JAK3*** | 218 | ***NRAS*** | 15 | ***SPOP*** | 186 |
| ***BCL2*** | 597 | ***EPHA3*** | 130 | ***KDM5C*** | 158 | ***NTRK1*** | 12 | ***SRC*** | 306 |
| ***BCOR*** | 91 | ***EPHA5*** | 142 | ***KDM6A*** | 130 | ***NTRK3*** | 51 | ***STAG2*** | 88 |
| ***BRAF*** | 7 | ***ERBB2*** | 87 | ***KDR*** | 16 | ***PALB2*** | 168 | ***STK11*** | 65 |
| ***BRCA1*** | 118 | ***ERBB3*** | 160 | ***KEAP1*** | 160 | ***PAX5*** | 353 | ***SUFU*** | 183 |
| ***BRCA2*** | 76 | ***ERBB4*** | 64 | ***KIT*** | 16 | ***PBRM1*** | 117 | ***TET2*** | 182 |
| ***BTK*** | 111 | ***ESR1*** | 388 | ***KRAS*** | 252 | ***PDGFRA*** | 53 | ***TGFBR2*** | 119 |
| ***CARD11*** | 39 | ***EZH2*** | 111 | ***LRP1B*** | 15 | ***PDGFRB*** | 76 | ***TNFAIP3*** | 222 |
| ***CBL*** | 27 | ***FAM123B*** | 50 | ***MAP2K1*** | 29 | ***PIK3CA*** | 88 | ***TOP1*** | 50 |
| ***CCND1*** | 418 | ***FANCA*** | 35 | ***MAP2K2*** | 13 | ***PIK3CG*** | 87 | ***TP53*** | 25 |
| ***CCND2*** | 36 | ***FANCD2*** | 51 | ***MAP2K4*** | 191 | ***PIK3R1*** | 23 | ***TSC1*** | 160 |
| ***CCND3*** | 683 | ***FBXW7*** | 88 | ***MAP3K1*** | 87 | ***PPP2R1A*** | 27 | ***TSC2*** | 76 |
| ***CCNE1*** | 186 | ***FGFR1*** | 71 | ***MCL1*** | 273 | ***PRDM1*** | 45 | ***TSHR*** | 182 |
| ***CD79A*** | 83 | ***FGFR2*** | 100 | ***MDM2*** | 385 | ***PTCH1*** | 194 | ***VHL*** | 130 |
| ***CDC73*** | 1274 | ***FGFR3*** | 37 | ***MED12*** | 26 | ***PTEN*** | 20 | ***WT1*** | 132 |
| ***CDH1*** | 131 | ***FGFR4*** | 136 | ***MEN1*** | 128 | ***PTPN11*** | 174 | ***XPO1*** | 101 |
| ***CDK12*** | 40 | ***FLT1*** | 71 | ***MET*** | 50 | ***RAD51*** | 176 |  |  |

**Table S3. *Adjusted* gene mutation value for each gene for lung cancer**

| ***ABL1*** | 137 | ***CDK12*** | 32 | ***FLT1*** | 24 | ***MLH1*** | 85 | ***RAD51*** | 61 |
| --- | --- | --- | --- | --- | --- | --- | --- | --- | --- |
| ***AKT1*** | 89 | ***CDK4*** | 25 | ***FLT3*** | 45 | ***MLL*** | 45 | ***RAF1*** | 99 |
| ***AKT2*** | 52 | ***CDK6*** | 228 | ***FLT4*** | 116 | ***MLL2*** | 16 | ***RARA*** | 27 |
| ***AKT3*** | 35 | ***CDKN1B*** | 67 | ***GATA1*** | 61 | ***MPL*** | 171 | ***RB1*** | 18 |
| ***ALK*** | 40 | ***CDKN2A*** | 17 | ***GATA2*** | 87 | ***MSH2*** | 116 | ***RET*** | 51 |
| ***APC*** | 45 | ***CHEK1*** | 41 | ***GATA3*** | 32 | ***MSH6*** | 100 | ***RICTOR*** | 47 |
| ***AR*** | 104 | ***CHEK2*** | 23 | ***GNAQ*** | 94 | ***MTOR*** | 45 | ***RNF43*** | 16 |
| ***ARAF*** | 45 | ***CIC*** | 271 | ***GNAS*** | 11 | ***MUTYH*** | 11 | ***RPTOR*** | 59 |
| ***ARID1A*** | 14 | ***CREBBP*** | 21 | ***HRAS*** | 109 | ***MYD88*** | 50 | ***RUNX1*** | 63 |
| ***ARID2*** | 27 | ***CSF1R*** | 70 | ***IDH1*** | 83 | ***NF1*** | 29 | ***SETD2*** | 9 |
| ***ASXL1*** | 139 | ***CTCF*** | 81 | ***IDH2*** | 46 | ***NF2*** | 94 | ***SF3B1*** | 46 |
| ***ATM*** | 20 | ***CTNNB1*** | 14 | ***IGF1R*** | 58 | ***NFE2L2*** | 20 | ***SMAD2*** | 51 |
| ***ATR*** | 41 | ***DAXX*** | 111 | ***IL7R*** | 83 | ***NKX2-1*** | 45 | ***SMAD4*** | 16 |
| ***ATRX*** | 24 | ***DDR2*** | 77 | ***JAK1*** | 67 | ***NOTCH1*** | 40 | ***SMARCA4*** | 10 |
| ***AURKB*** | 89 | ***DNMT3A*** | 44 | ***JAK2*** | 35 | ***NOTCH2*** | 51 | ***SMARCB1*** | 228 |
| ***AXL*** | 40 | ***EGFR*** | 7 | ***JAK3*** | 101 | ***NPM1*** | 48 | ***SMO*** | 51 |
| ***BAP1*** | 20 | ***EP300*** | 359 | ***KDM5C*** | 91 | ***NRAS*** | 198 | ***SPEN*** | 31 |
| ***BCL2*** | 40 | ***EPHA3*** | 17 | ***KDM6A*** | 81 | ***NTRK1*** | 55 | ***SPOP*** | 10 |
| ***BCOR*** | 47 | ***EPHA5*** | 35 | ***KDR*** | 29 | ***NTRK3*** | 83 | ***SRC*** | 472 |
| ***BRAF*** | 22 | ***ERBB2*** | 24 | ***KEAP1*** | 33 | ***PALB2*** | 87 | ***STAG2*** | 89 |
| ***BRCA1*** | 66 | ***ERBB3*** | 87 | ***KIT*** | 51 | ***PAX5*** | 290 | ***STK11*** | 15 |
| ***BRCA2*** | 42 | ***ERBB4*** | 40 | ***KRAS*** | 10 | ***PBRM1*** | 55 | ***SUFU*** | 54 |
| ***BTK*** | 33 | ***ESR1*** | 212 | ***LRP1B*** | 29 | ***PDGFRA*** | 57 | ***TET2*** | 77 |
| ***CARD11*** | 31 | ***EZH2*** | 166 | ***MAP2K1*** | 133 | ***PDGFRB*** | 20 | ***TGFBR2*** | 175 |
| ***CBL*** | 46 | ***FAM123B*** | 33 | ***MAP2K2*** | 139 | ***PIK3CA*** | 17 | ***TNFAIP3*** | 51 |
| ***CCND1*** | 104 | ***FANCA*** | 47 | ***MAP2K4*** | 212 | ***PIK3CG*** | 51 | ***TOP1*** | 20 |
| ***CCND2*** | 404 | ***FANCD2*** | 33 | ***MAP3K1*** | 79 | ***PIK3R1*** | 76 | ***TP53*** | 11 |
| ***CCNE1*** | 39 | ***FBXW7*** | 62 | ***MDM2*** | 99 | ***PPP2R1A*** | 31 | ***TSC1*** | 133 |
| ***CD79A*** | 228 | ***FGFR1*** | 122 | ***MED12*** | 41 | ***PRDM1*** | 68 | ***TSC2*** | 14 |
| ***CD79B*** | 109 | ***FGFR2*** | 34 | ***MEN1*** | 88 | ***PTCH1*** | 44 | ***TSHR*** | 112 |
| ***CDC73*** | 45 | ***FGFR3*** | 61 | ***MET*** | 18 | ***PTEN*** | 116 | ***WT1*** | 45 |
| ***CDH1*** | 137 | ***FGFR4*** | 86 | ***MITF*** | 54 | ***PTPN11*** | 212 | ***XPO1*** | 41 |

**Table S4.** **Melanoma cohort treated with ipilimumab (n=76)**

| **Patient No.** | **PTML** | **Age** | **Gender** | **M stage** | **Subtype** | **LDH** | **OS (days)** | **OS (status)** |
| --- | --- | --- | --- | --- | --- | --- | --- | --- |
| 1 | Low | 44 | Female | M1b | Unknown | Elevated | 78 | Dead |
| 2 | Low | 73 | Male | M1b | Cutaneous | Not Elevated | 582 | Dead |
| 3 | Low | 54 | Female | M1b | Cutaneous | Not Elevated | 890 | Dead |
| 4 | Low | 48 | Male | M1a | Unknown | Not Elevated | 692 | Alive |
| 5 | Low | 37 | Female | M1a | Unknown | Not Elevated | 95 | Alive |
| 6 | Low | 56 | Male | M1c | Cutaneous | Not Elevated | 169 | Dead |
| 7 | Low | 43 | Male | M1a | Cutaneous | Not Elevated | 147 | Alive |
| 8 | Low | 59 | Male | M1c | Cutaneous | Not Elevated | 194 | Dead |
| 9 | Low | 60 | Female | M1a | Cutaneous | Not Elevated | 212 | Alive |
| 10 | Low | 47 | Female | M1a | Cutaneous | Elevated | 58 | Dead |
| 11 | Low | 41 | Male | M1c | Unknown | Elevated | 383 | Dead |
| 12 | Low | 60 | Male | M1c | Cutaneous | Elevated | 450 | Dead |
| 13 | Low | 43 | Female | M1c | Cutaneous | Not Elevated | 419 | Dead |
| 14 | Low | 64 | Male | M1c | Unknown | Not Elevated | 853 | Alive |
| 15 | Low | 54 | Male | M1a | Cutaneous | Not Elevated | 59 | Alive |
| 16 | Low | 42 | Female | M1c | Cutaneous | Not Elevated | 473 | Alive |
| 17 | Low | 73 | Male | M1c | Cutaneous | Not Elevated | 676 | Alive |
| 18 | Low | 69 | Male | M1a | Cutaneous | Not Elevated | 336 | Dead |
| 19 | Low | 48 | Female | M1c | Cutaneous | Not Elevated | 1487 | Alive |
| 20 | High | 40 | Female | M1a | Cutaneous | Elevated | 622 | Alive |
| 21 | High | 44 | Female | M1b | Cutaneous | Not Elevated | 391 | Alive |
| 22 | High | 41 | Male | M1b | Cutaneous | Not Elevated | 236 | Dead |
| 23 | High | 75 | Male | M1c | Unknown | Not Elevated | 236 | Dead |
| 24 | High | 69 | Male | M1c | Cutaneous | Not Elevated | 415 | Alive |
| 25 | High | 64 | Male | M1c | Cutaneous | Not Elevated | 202 | Alive |
| 26 | High | 43 | Male | M1b | Cutaneous | Not Elevated | 476 | Alive |
| 27 | High | 73 | Female | M1a | Cutaneous | Not Elevated | 635 | Alive |
| 28 | High | 55 | Female | M1c | Cutaneous | Not Elevated | 230 | Dead |
| 29 | High | 72 | Male | M1a | Cutaneous | Not Elevated | 664 | Dead |
| 30 | High | 18 | Female | M1c | Cutaneous | Elevated | 248 | Dead |
| 31 | High | 41 | Male | M1c | Cutaneous | Not Elevated | 1176 | Alive |
| 32 | High | 32 | Male | M1a | Cutaneous | Not Elevated | 395 | Dead |
| 33 | High | 76 | Male | M1b | Cutaneous | Not Elevated | 144 | Dead |
| 34 | High | 65 | Male | M1a | Unknown | Elevated | 402 | Alive |
| 35 | High | 66 | Male | M1c | Unknown | Not Elevated | 576 | Alive |
| 36 | High | 54 | Male | M1c | Cutaneous | Not Elevated | 1288 | Alive |
| 37 | High | 39 | Female | M1c | Cutaneous | Elevated | 914 | Dead |
| 38 | High | 22 | Female | M1c | Cutaneous | Not Elevated | 2476 | Alive |
| 39 | High | 63 | Male | M1b | Unknown | Not Elevated | 1253 | Alive |
| 40 | High | 47 | Female | M1c | Cutaneous | Not Elevated | 579 | Alive |
| 41 | High | 63 | Male | M1b | Cutaneous | Not Elevated | 197 | Alive |
| 42 | High | 66 | Male | M1a | Cutaneous | Not Elevated | 194 | Alive |
| 43 | High | 54 | Male | M1b | Unknown | Not Elevated | 1292 | Alive |
| 44 | High | 77 | Female | M1a | Cutaneous | Elevated | 487 | Dead |
| 45 | High | 67 | Male | M1c | Cutaneous | Elevated | 605 | Dead |
| 46 | High | 55 | Male | M1b | Cutaneous | Not Elevated | 628 | Alive |
| 47 | High | 43 | Male | M1b | Cutaneous | Not Elevated | 948 | Alive |
| 48 | High | 59 | Male | M1c | Cutaneous | Not Elevated | 894 | Alive |
| 49 | High | 64 | Male | M1a | Cutaneous | Not Elevated | 520 | Alive |
| 50 | High | 30 | Female | M1c | Cutaneous | Not Elevated | 1170 | Alive |
| 51 | High | 52 | Male | M1c | Cutaneous | Not Elevated | 829 | Alive |
| 52 | High | 68 | Male | M1c | Unknown | Not Elevated | 479 | Alive |
| 53 | High | 40 | Female | M1c | Cutaneous | Not Elevated | 227 | Dead |
| 54 | High | 71 | Male | M1c | Unknown | Elevated | 281 | Alive |
| 55 | High | 68 | Male | M1c | Cutaneous | Not Elevated | 258 | Dead |
| 56 | High | 62 | Male | M1b | Cutaneous | Not Elevated | 931 | Alive |
| 57 | High | 34 | Male | M1c | Unknown | Not Elevated | 1148 | Alive |
| 58 | High | 70 | Male | M1a | Cutaneous | Not Elevated | 1547 | Alive |
| 59 | High | 72 | Male | M1c | Cutaneous | Not Elevated | 572 | Alive |
| 60 | High | 52 | Male | M1b | Cutaneous | Not Elevated | 942 | Alive |
| 61 | High | 59 | Male | M1a | Unknown | Elevated | 835 | Dead |
| 62 | High | 69 | Male | Unknown | Cutaneous | Not Elevated | 499 | Dead |
| 63 | High | 60 | Male | M1b | Cutaneous | Not Elevated | 453 | Alive |
| 64 | High | 65 | Male | M1b | Cutaneous | Not Elevated | 392 | Alive |
| 65 | High | 76 | Male | M1a | Unknown | Not Elevated | 436 | Alive |
| 66 | High | 62 | Male | M1c | Cutaneous | Elevated | 490 | Dead |
| 67 | High | 56 | Male | M1c | Cutaneous | Elevated | 92 | Alive |
| 68 | High | 74 | Male | M1c | Cutaneous | Not Elevated | 490 | Alive |
| 69 | High | 61 | Male | M1c | Unknown | Not Elevated | 528 | Alive |
| 70 | High | 63 | Male | M1b | Cutaneous | Not Elevated | 718 | Alive |
| 71 | High | 75 | Male | M1b | Unknown | Not Elevated | 703 | Alive |
| 72 | High | 64 | Male | M1c | Cutaneous | Not Elevated | 610 | Alive |
| 73 | High | 67 | Male | M1c | Cutaneous | Not Elevated | 733 | Alive |
| 74 | High | 69 | Male | Unknown | Unknown | Not Elevated | 1231 | Dead |
| 75 | High | 53 | Male | M1c | Unknown | Not Elevated | 362 | Alive |
| 76 | High | 51 | Male | M1a | Cutaneous | Not Elevated | 215 | Alive |

**Table S5.** **Melanoma cohort treated with ACT-TIL (n=36)**

1. **Supplementary Figures**

**Figure S1. Derivation of the PTML**


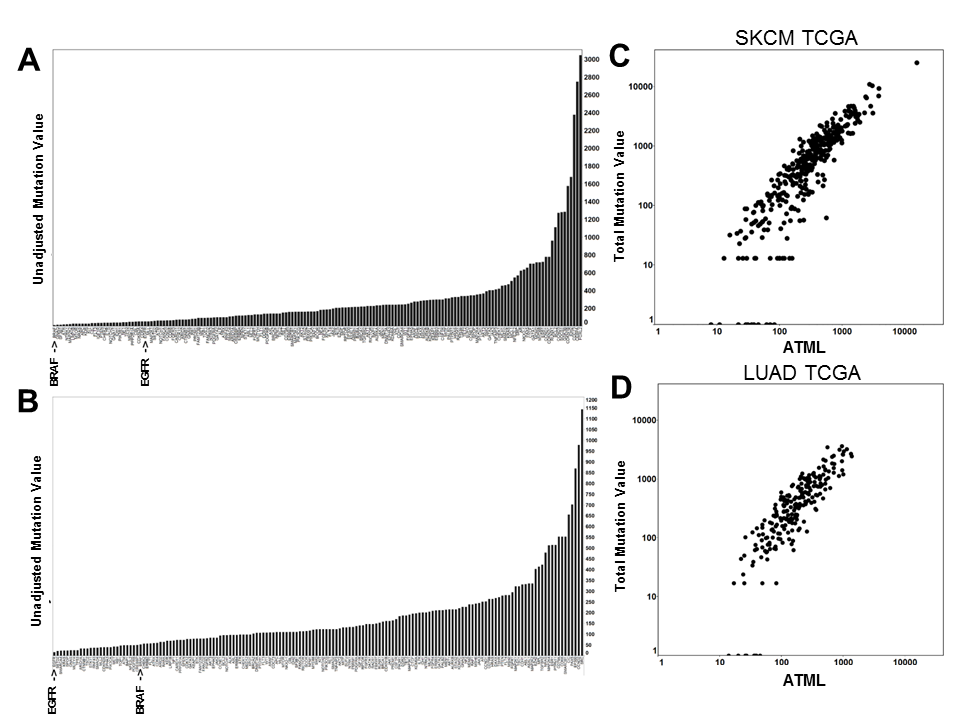


A) Melanoma (SKCM) TCGA (n = 345) and B) Lung (LUAD) TCGA (n = 230) samples were interrogated for non-synonymous exonic mutations in a set of 170 genes used in clinical NGS panels. Genes with at least one mutation are plotted on the x-axis relative to their corresponding unadjusted gene mutation values. The differences in the unadjusted mutation values given to BRAF versus EGFR mutations within the SKCM versus LUAD TCGA data sets are indicated. For the C) SKCM and D) LUAD TCGA datasets, the total mutation value for each sample (sum of the unadjusted gene mutation values for each sample) are plotted on the y-axis and the ATML within each sample, as determined by WES, are plotted on the x-axis. R^2^ values were 0.87 for SKCM and 0.76 for LUAD. PTML, Predicted Total Mutation Load; SKCM, cutaneous melanoma samples; TCGA, The Cancer Genome Atlas; LUAD, lung adenocarcinoma samples; NGS, next generation sequencing; ATML, Actual Total Mutation Load; WES, whole exome sequencing.

**Figure S2. Cancer Specificity of PTML**


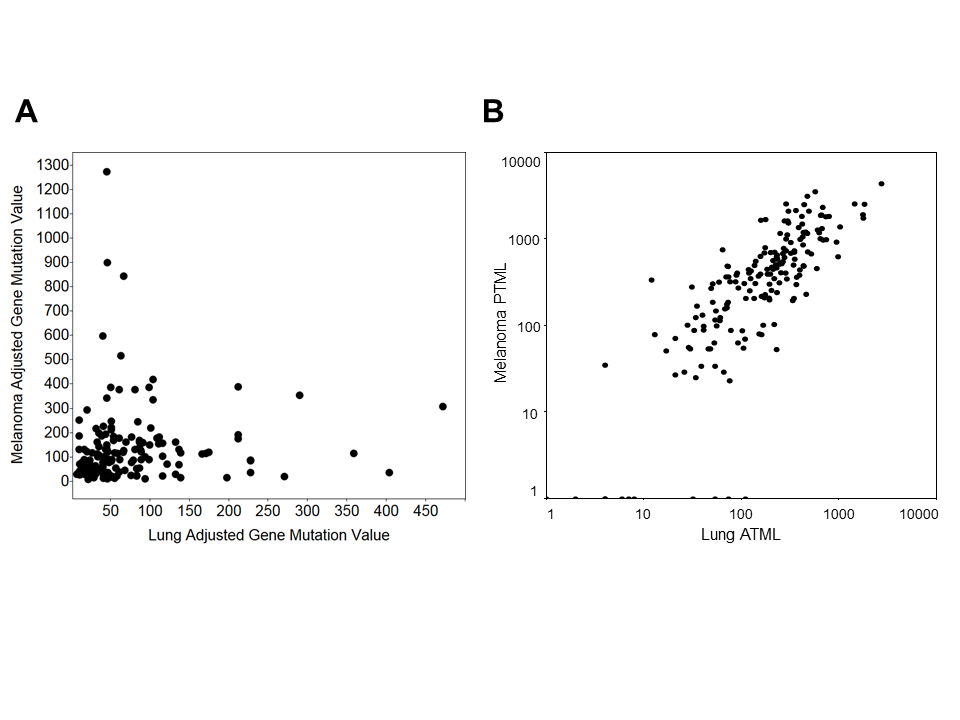


A) Application of the Melanoma Adjusted Gene Mutaiton Value to the lung Adjusted Gene Mutation Value shows low correlation (R^2^ = 0.0063).

B) Application of the melanoma-specific PTML to the lung cancer ATML validation set shows low correlation (R^2^ = 0.47).

**Figure S3. Total mutation burden predicted using recurrently mutated genes**

**
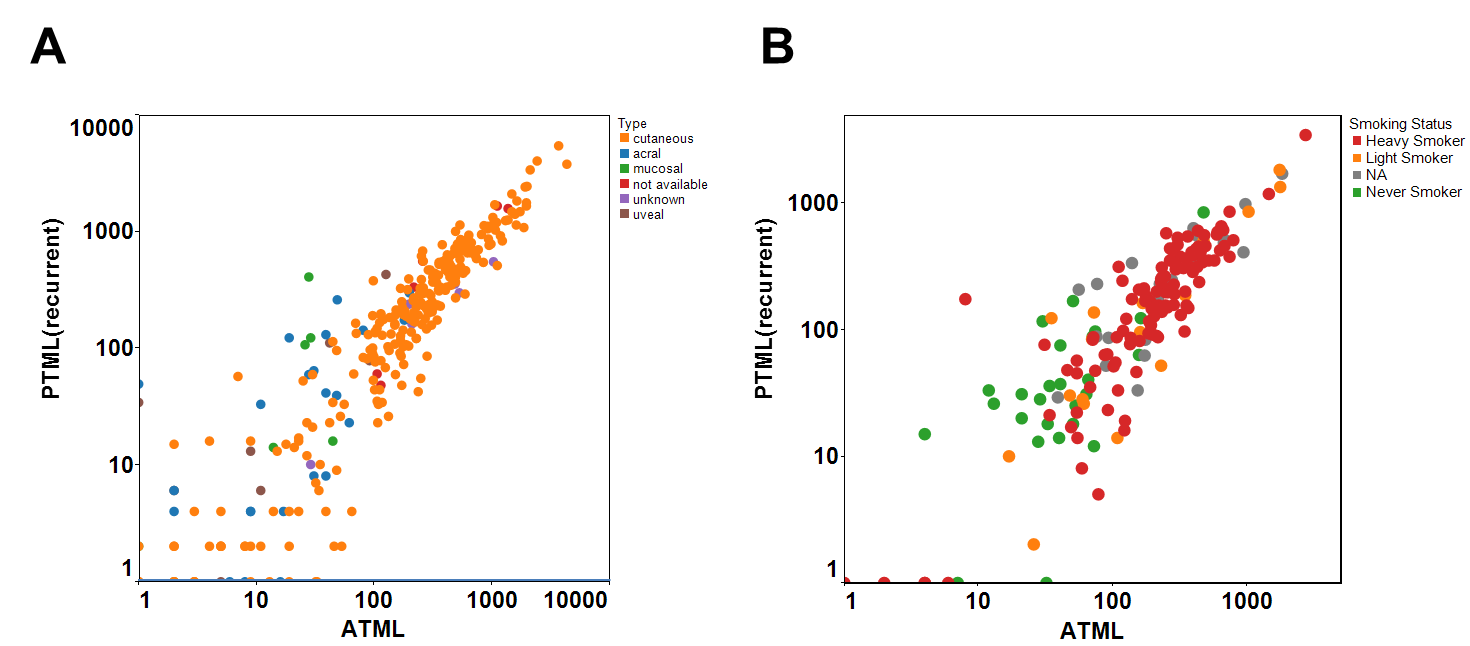
**

A) The most recurrent somatically mutated genes within the SKCM TCGA were used to generate a predicted total mutation load, PTML(recurrent), for melanoma samples derived from three independent cohorts [1-3]. PTML(recurrent) versus ATML values for each melanoma sample are shown (R^2^ = 0.879 for cutaneous melanomas, n = 258; R^2^=0.878 for all melanoma subtypes, n=312). B) The most recurrent somatically mutated genes within the LUAD TCGA were used to generate a predicted total mutation load, PTML(recurrent), for lung cancer samples [4]. PTML(recurrent) versus ATML values for each lung cancer sample are shown (R^2^ = 0.878, n = 182).

**Figure S4. PTML correlates with characteristic nucleotide types**


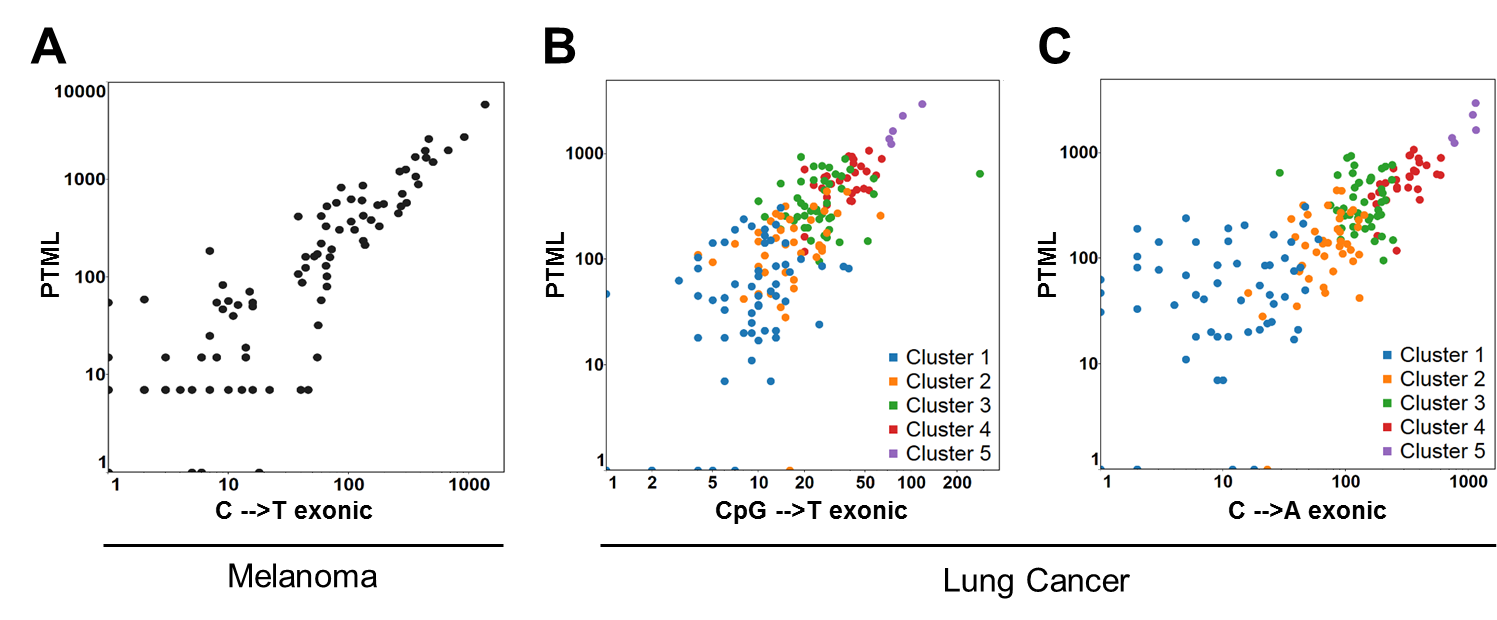


Analysis of PTML (y-axis) vs. A) C → T exonic transitions (x-axis, a defining UV-mediated nucleotide alteration) for each sample in the skin melanoma cohort (n = 127) [2]; B) CpG → T transitions or C) C → A transversions (x-axis, defining tobacco-mediated nucleotide alterations) for each sample in the lung cancer cohort (n = 183) [4]. Imielinski et al [4] performed unbiased hierarchical clustering of context-specific mutation rates across 183 lung cancer samples resulting in five mutation spectrum clusters ranging from low mutation “never-smoker” samples (Cluster 1, blue) to hyper-mutated samples (Cluster 5, purple). Clusters 2-4 represent varying degrees of tobacco-induced nucleotide alterations in the samples. The hierarchical clusters demonstrate an ascending order relative to the PTML score.

**Figure S5. Low PTML correlates with poorer clinical results in anti-CTLA-4 treated melanoma tumors**


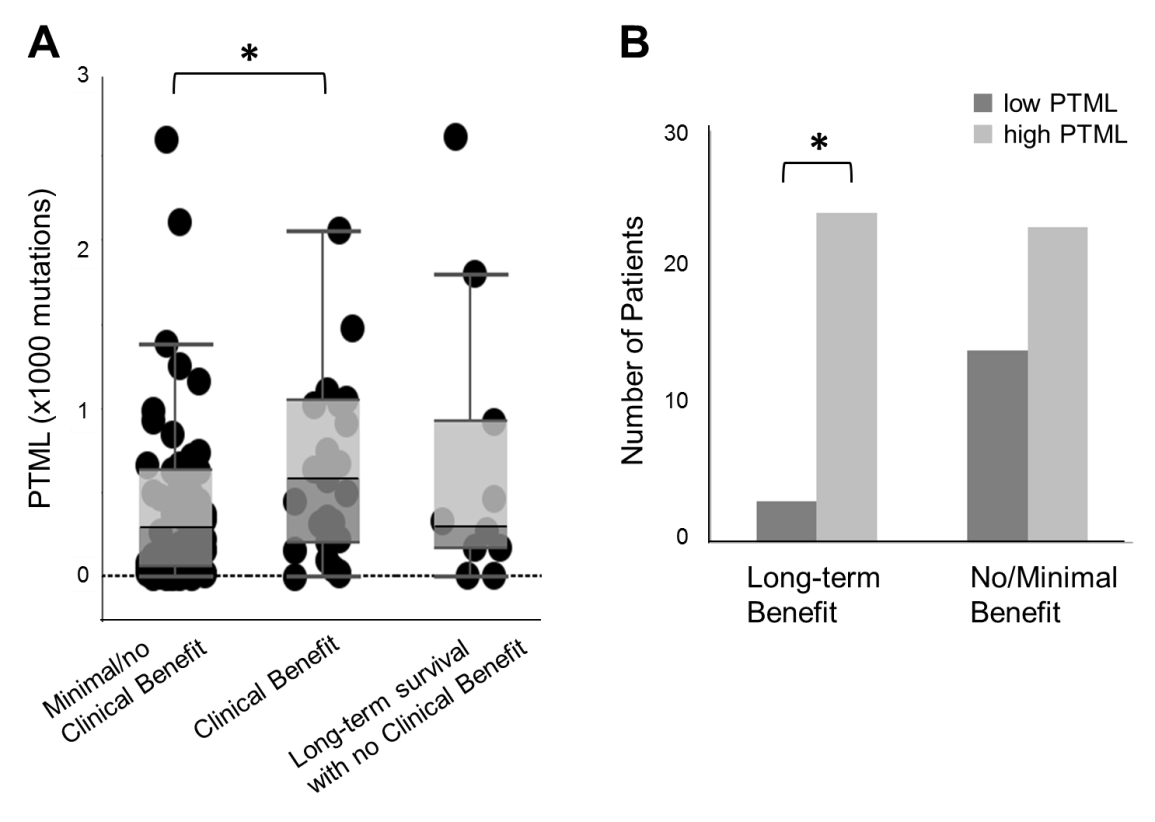


A) The anti-CTLA-4 treated melanoma cohort was grouped by clinical outcome as previously described: clinical benefit (n = 27), minimal or no clinical benefit (n = 73) or long survival without clinical benefit (n = 10), and comparison of PTML between the groups was determined [6]. Patients with clinical benefit had significantly higher PTML than those who had minimal or no clinical benefit (*P* = .006). An additional 5 points are not shown because of outlying high mutation loads in 2 minimal/no clinical benefit and 3 clinical benefit patients. B) A distinct anti-CTLA-4 treated melanoma cohort was grouped by clinical outcome as previously described: Long-term benefit (n = 27) or No/Minimal Benefit (n = 37), and comparison between low PTML (≤ 100) and high PTML (> 100) groups was determined [3]. Significantly fewer low PTML patients (n = 3) achieved long-term benefit (LTB) as opposed to high PTML patients (n = 24) (*P* < .05). Fourteen low PTML patients had no or minimal benefit (NMB) as opposed to 23 high PTML patients.

1. **Supplementary References**
2. Hodis E, Watson IR, Kryukov GV, Arold ST, Imielinski M, Theurillat JP, Nickerson E, Auclair D, Li L, Place C *et al*: **A landscape of driver mutations in melanoma.** Cell 2012, **150**(2):251-263.
3. Krauthammer M, Kong Y, Ha BH, Evans P, Bacchiocchi A, McCusker JP, Cheng E, Davis MJ, Goh G, Choi M *et al*: **Exome sequencing identifies recurrent somatic RAC1 mutations in melanoma.** Nature genetics 2012, **44**(9):1006-1014.
4. Snyder A, Makarov V, Merghoub T, Yuan J, Zaretsky JM, Desrichard A, Walsh LA, Postow MA, Wong P, Ho TS *et al*: **Genetic basis for clinical response to CTLA-4 blockade in melanoma.** The New England journal of medicine 2014, **371**(23):2189-2199.
5. Imielinski M, Berger AH, Hammerman PS, Hernandez B, Pugh TJ, Hodis E, Cho J, Suh J, Capelletti M, Sivachenko A *et al*: **Mapping the hallmarks of lung adenocarcinoma with massively parallel sequencing.** Cell 2012, **150**(6):1107-1120.
6. Rizvi NA, Hellmann MD, Snyder A, Kvistborg P, Makarov V, Havel JJ, Lee W, Yuan J, Wong P, Ho TS *et al*: **Mutational landscape determines sensitivity to PD-1 blockade in non-small cell lung cancer.** Science 2015; **348**(6230):124-8.
7. Van Allen EM, Miao D, Schilling B, Shukla SA, Blank C, Zimmer L, Sucker A, Hillen U, Geukes Foppen MH, Goldinger SM *et al*. **Genomic correlates of response to CTLA-4 blockade in metastatic melanoma.** Science. 2015;**350**:207-11.
